# Reply to: Association between rectus femoris cross-sectional area and diaphragmatic excursion with weaning of tracheostomized patients in the intensive care unit

**DOI:** 10.62675/2965-2774.20240012-en

**Published:** 2024-04-26

**Authors:** Fernando Nataniel Vieira, Raquel Bortoluzzi Bertazzo, Gabriela Carvalho Nascimento, Mariluce Anderle, Ana Cláudia Coelho, Fabiana de Oliveira Chaise, Jaqueline da Silva Fink, Wagner Luis Nedel, Bruna Ziegler

**Affiliations:** 2 Universidade Federal do Rio Grande do Sul Porto Alegre RS Brazil Postgraduate Program in Pulmonological Sciences, Universidade Federal do Rio Grande do Sul - Porto Alegre (RS), Brazil.; 2 Grupo Hospitalar Conceição Intensive Care Unit Porto Alegre RS Brazil Intensive Care Unit, Grupo Hospitalar Conceição - Porto Alegre (RS), Brazil.; 3 Universidade Federal do Rio Grande do Sul Hospital de Clínicas de Porto Alegre Physiotherapy Unit Porto Alegre RS Brazil Physiotherapy Unit, Hospital de Clínicas de Porto Alegre, Universidade Federal do Rio Grande do Sul - Porto Alegre (RS), Brazil.

**Dear editor,**

We appreciate the interesting and thoughtful critique provided by Finsterer et al. in the letter to the editor regarding our article titled "Association between rectus femoris cross-sectional area and diaphragmatic excursion with weaning of tracheostomized patients in the intensive care unit".^(1)^ We would like to address the raised concerns and provide additional clarification on certain aspects of our study.

It is important to note that there are inherent conceptual issues regarding conducting an observational clinical study. Our cohort is a representative sample of critically ill patients undergoing prolonged weaning from mechanical ventilation, in accordance with best practices in this field. In these patients, successful ventilatory weaning depends on several variables, including diaphragmatic function. In addition, our results suggest an association between peripheral muscle area and successful ventilatory weaning. No existing models in the literature can adequately predict the probability of a patient progressing to the need for prolonged ventilation^(2)^ owing to the clinical and pathophysiological variability that contributes to this clinical condition.

We recognize that multiple factors influence the cross-sectional area of the rectus femoris and diaphragmatic excursion, posing challenges in standardizing these measurements. On the other hand, the adaptability of these variables in response to specific clinical conditions prompts consideration of whether they can effectively function as outcome indicators in this population. Considering potentially confounding variables impacting the outcome (ventilatory success) postulated by Finsterer et al., many of which are challenging to measure in clinical practice, we performed a multivariate analysis using logistic regression to evaluate the association between the measurements and the outcome. Multivariate analysis can show which of the various factors being assessed has the strongest association with an outcome and provides a measure of the magnitude of the potential influence.^(3)^ It also has the ability to "adjust" for confounding factors, i.e., factors that are associated with both other predictor variables and the outcome, so the measure of the influence of the predictor of interest is not distorted by the effect of the confounder.^(3)^ An article previously published in this journal provides important recommendations regarding the critical reading of cohort studies.^(4)^

We acknowledge that our study is limited by its single-center design. However, it is important to note that the majority of studies in this field are unicentric^(5)^ or, at most, have studied populations from a few centers.^(6)^ Our study focused on a very specific but clinically relevant population (chronic critically ill patients ventilated by tracheostomy), which justifies the single-center approach that is commonly adopted in this area. This is an area of research that is still very promising, with several gaps to be addressed in new studies. Collaborative studies in this area are necessary and welcome.
