# Supplementary material for: Reply to: Association between rectus femoris cross-sectional area and diaphragmatic excursion with weaning of tracheostomized patients in the intensive care unit
Source: Crit Care Sci. 2024 Apr 26;36:e20240012en. doi: 10.62675/2965-2774.20240012-en (PMC11132113; doi:10.62675/2965-2774.20240012-en)
Supplement: Translation

# Translation: Resposta para: Associação entre área de secção transversa do reto femoral e excursão diafragmática com o desmame de pacientes com traqueostomia na unidade de terapia intensiva

**DOI:** 10.62675/2965-2774.20240012-pt

**Authors:** Fernando Nataniel Vieira, Raquel Bortoluzzi Bertazzo, Gabriela Carvalho Nascimento, Mariluce Anderle, Ana Cláudia Coelho, Fabiana de Oliveira Chaise, Jaqueline da Silva Fink, Wagner Luis Nedel, Bruna Ziegler

**Affiliations:** 1 Porto Alegre RS Brasil Programa de Pós-Graduação em Ciências Pneumológicas, Universidade Federal do Rio Grande do Sul - Porto Alegre (RS), Brasil.; 2 Porto Alegre RS Brasil Unidade de Terapia Intensiva, Grupo Hospitalar Conceição - Porto Alegre (RS), Brasil.; 6 Porto Alegre RS Brasil Unidade de Fisioterapia, Hospital de Clínicas de Porto Alegre, Universidade Federal do Rio Grande do Sul - Porto Alegre (RS), Brasil.

**Prezado editor,**

Agradecemos a crítica interessante e atenciosa fornecida por Finsterer et al. na carta ao editor sobre nosso artigo intitulado "Associação entre área de secção transversa do reto femoral e excursão diafragmática com o desmame de pacientes com traqueostomia na unidade de terapia intensiva".^(1)^ Gostaríamos de abordar as preocupações levantadas e fornecer esclarecimentos adicionais sobre determinados aspectos de nosso estudo.

Vale ressaltar que existem questões conceituais inerentes à realização de um estudo clínico observacional. Nossa coorte é uma amostra representativa de pacientes graves submetidos a desmame prolongado da ventilação mecânica, de acordo com as melhores práticas nesse campo. Nesses pacientes, o sucesso do desmame ventilatório depende de diversas variáveis, inclusive da função diafragmática. Além disso, nossos resultados sugerem haver uma associação entre a área muscular periférica e o sucesso do desmame ventilatório. Nenhum modelo existente na literatura pode prever adequadamente a probabilidade de um paciente evoluir para a necessidade de ventilação prolongada^(2)^ devido à variabilidade clínica e fisiopatológica que contribui para essa condição clínica.

Reconhecemos que vários fatores influenciam a área da secção transversa do reto femoral e a excursão diafragmática, o que representa um desafio para a padronização dessas medidas. Por outro lado, a adaptabilidade dessas variáveis em resposta a condições clínicas específicas leva a considerar se elas podem efetivamente funcionar como indicadores de desfecho nessa população. Considerando as possíveis variáveis de confusão que afetam o desfecho (sucesso ventilatório) postulado por Finsterer et al., muitas das quais são difíceis de medir na prática clínica, realizamos uma análise multivariada usando regressão logística para avaliar a associação entre as medidas e o desfecho. A análise multivariada pode mostrar qual dos vários fatores que estão sendo avaliados está mais fortemente associado a um desfecho e fornece uma medida da magnitude da influência potencial.^(3)^ Ela também tem a capacidade de "ajustar" os fatores de confusão, ou seja, fatores que estão associados a outras variáveis preditoras e ao desfecho, de modo que a medida da influência do preditor de interesse não seja distorcida pelo efeito do fator de confusão.^(3)^ Um artigo publicado anteriormente nesta revista fornece recomendações importantes sobre a leitura crítica de estudos de coorte.^(4)^

Reconhecemos que nosso estudo é limitado por seu desenho unicêntrico. No entanto, é importante observar que a maioria dos estudos nesse campo são unicêntricos^(5)^ ou, no máximo, estudou populações de alguns centros.^(6)^ Nosso estudo concentrou-se em uma população muito específica, mas clinicamente relevante (pacientes críticos crônicos ventilados por traqueostomia), o que justifica a abordagem unicêntrica que é comumente adotada nessa área. Essa é uma área de pesquisa ainda bastante promissora, com várias lacunas a serem abordadas em novos estudos. Estudos colaborativos nessa área são necessários e bem-vindos.
